# Evaluation of monoxide film-based dosimeters for surface dose detection in electron therapy

**DOI:** 10.1371/journal.pone.0251441

**Published:** 2021-05-21

**Authors:** Moo Jae Han, Seung Woo Yang, Sang Il Bae, Young Min Moon, Wan Jeon, Chul Won Choi, Sung Kwang Park, Jin Young Kim

**Affiliations:** 1 Department of Radiation Oncology, College of Medicine, Inje University, Busan, Republic of Korea; 2 Departments of Radiation Oncology, Dongnam Institute of Radiological and Medical Sciences, Busan, Republic of Korea; 3 Department of Radiation Oncology, Busan Paik Hospital, Inje University, Busan, Republic of Korea; University of Seville, SPAIN

## Abstract

Generally, electron therapy is applied to tumors on or close to the skin surface. However, this causes a variety of skin-related side effects. To alleviate the risk of these side effects, clinical treatment uses skin dosimeters to verify the therapeutic dose. However, dosimeters suffer from poor accuracy, because their attachment sites are approximated with the help of naked eyes. Therefore, a dosimeter based on a flexible material that can adjust to the contours of the human body is required. In this study, the reproducibility, linearity, dose-rate dependence, and percentage depth ionization (PDI) of PbO and HgO film-based dosimeters are evaluated to explore their potential as large-scale flexible dosimeters. The results demonstrate that both dosimeters deliver impressive reproducibility (within 1.5%) and linearity (≥ 0.9990). The relative standard deviations of the dose-rate dependence of the PbO and HgO dosimeters were 0.94% and 1.16% at 6 MeV, respectively, and 1.08% and 1.25% at 9 MeV, respectively, with the PbO dosimeter outperforming the 1.1% of existing diodes. The PDI analysis of the PbO and HgO dosimeters returned values of 0.014 cm (–0.074 cm) and 0.051 cm (–0.016 cm), respectively at 6 MeV (9 MeV) compared to the thimble chamber and R_50_. Therefore, the maximum error of each dosimeter is within the allowable range of 0.1 cm. In short, the analysis reveals that the PbO dosimeter delivers a superior performance relative to its HgO counterpart and has strong potential for use as a surface dosimeter. Thus, flexible monoxide materials have the necessary qualities to be used for dosimeters that meet the requisite quality assurance standards and can satisfy a variety of radiation-related applications as flexible functional materials.

## Introduction

Electron beam therapy (EBT) has a short penetration depth, which makes it suitable for treating tumors close to the skin. The therapeutic dose of EBT is calculated on the basis of 80% or 90%, and the R_90_ at 6 MeV and 9 MeV corresponds to penetration depths of approximately 1.8 and 2.5 cm, respectively, where R_90_ denotes the clinical range defined as a depth of 90% relative dose [1].

Skin-related side effects, such as erythema, desquamation, necrosis, epitheliolysis, and hypohidrosis, are common in EBT patients [2–4]. However, since the accuracy of the skin dose calculated in the treatment planning system is only ± 20%, a skin dosimeter is used in clinical practice to verify the skin dose [5].

Commonly used dosimeters include films, glass dosimeters (GD), optically stimulated luminescent dosimeters (OSLDs), and thermo luminescent dosimeters (TLDs). However, none of these devices can obtain the dose distribution on the body surface because they measure the point dose with the integrated dosimeter of the analog detection method. Additionally, as the attachment part on a patient’s body, which is naturally curved, is checked visually, the positional accuracy can be unreliable [6]. For example, the average error rate of a digital MOSFET dosimeter was reported to be 22.8% [7]. Therefore, in clinical practice, there is an urgent demand for a patch-type digital surface dosimeter that can be attached to a patient’s skin to measure the body surface in real time.

A significant amount of radiation detector research is focused on developing flexible functional photoconductor materials [8,9]. Among the materials investigated thus far, lead oxide (PbO) and mercury oxide (HgO) have excellent physical properties with high atomic numbers (Z_Hg_: 80, Z_Pb_: 82, Z_O_: 8) and densities (ρ_PbO_: 9.53 g/cm^3^, ρ_HgO_: 11.14 g/cm^3^) [10–12]. Meanwhile, in the particle-in-binder (PIB) method, which involves mixing powder material and binder, flexible materials can be produced by using a silicone rubber binder. Additionally, the PIB method shows the possibility of improving the electrical stability via passivation of the material itself by using a binder with insulating properties. Therefore, as a basic study considering the development of a large-area surface dosimeter, this study focuses on evaluating the performance of monoxide materials fabricated under optimized manufacturing conditions.

In this study, flexible PbO and HgO dosimeters were manufactured to evaluate the LINAC quality assurance (QA) with respect to reproducibility, linearity, dose-rate dependence, and percentage depth ionization (PDI). Additionally, to verify the applicability of these dosimeters, they were compared with the measurement results of the diode and ion chamber.

## Experimental method

The PIB deposition method is popular in the field of radiation detectors because it is possible to manufacture functional materials according to the binder and to manufacture large areas easily [13]. Therefore, in this study, flexible unit cell sensors based on polycrystalline PbO and HgO materials manufactured by the PIB method were fabricated, with their performance compared and evaluated according to the radiation treatment QA requirements.

### Fabrication of the film dosimeters

**Fig 1:**
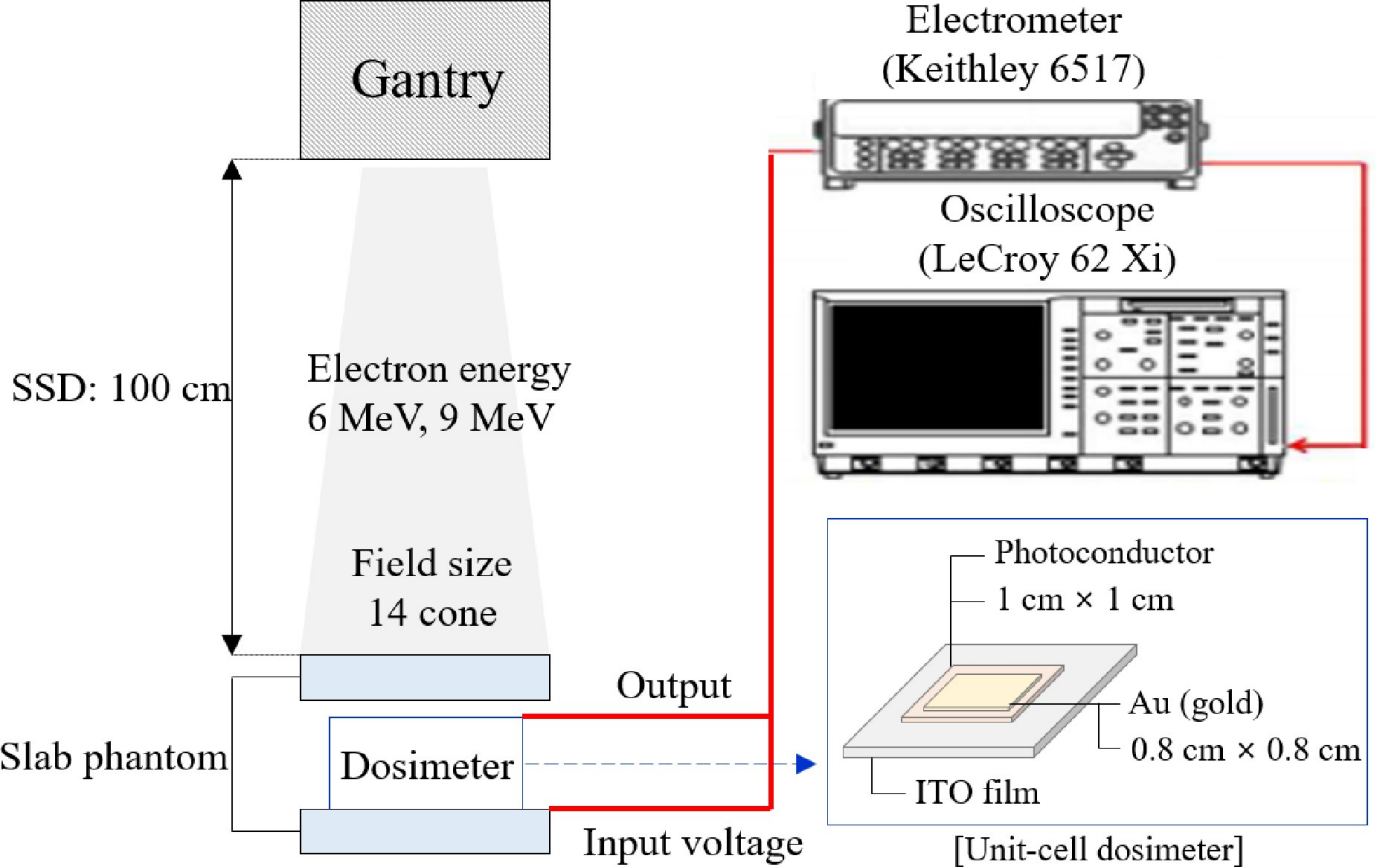
Schematic of the measurement setup.

A flexible indium tin oxide (ITO) film (polyester) substrate was used as the bottom electrode, with ultrasonic cleaning performed for 30 min to remove foreign substances [14]. Afterwards, the photoconductor material was prepared by mixing a T-2 binder (silicone rubber) with powdered PbO and HgO (Kojundo Chemical Laboratory Inc., Japan) with purities of 99.999% at a mixing ratio of 4:1. The mixed slurry-type photoconductor was applied using a screen-printing technique over an area of 1 cm × 1 cm. The thickness was 50 μm; to reduce this roughness to less than 5%, stone milling was performed for 30 min. And the material was dried in an oven at 70°C for 8 h. Additionally, to prevent oxidation-related changes to the physical properties, a 10 μm-thick passive layer was deposited on the photoconductor material using CVD deposition of C-type parylene. The top electrode, designed to collect the electric charge, was deposited with a purity of 99.999% gold (Sigma Aldrich Inc., USA) over an area of 0.8 cm × 0.8 cm on the material produced by the PVD method. Fig 1 shows a schematic diagram of the measurement setup used in this study.

To test the performance of the sensor, we evaluated the reproducibility and linearity of the sensor at 6 and 9 MeV. For the measurements, we used a LINAC system (Infinity, Elekta AB, Stockholm, Sweden) that can irradiate the sensor with cone-shaped beams. The source-to-surface distance (SSD) was set to 100 cm. The build-up material depth was set to 1.3 cm and 1.9 cm for the mean electron energies of 6 MeV and 9 MeV, respectively, by using a slab phantom (RW3, PTW, Freiburg, Germany).

The phantom provides a build-up region of “d-max” depth in which an electron equilibrium distribution is generated via stripped electrons, known as secondary electrons or δ-rays. An electrometer (Keithley, 6517A, USA) was used to apply a driving voltage of 1 V/μm through the fabricated sensor. Subsequently, an oscilloscope was used to acquire the waveforms of the radiation signals, while AcqKnowledge 4.2 software (Biopac, CANADA) was used to calculate the electrical charge collected from the acquired waveforms. Table 1 lists the irradiation conditions used during the measurement.

**Table 1:** Measurement conditions.

Nominal electron energy	6 MeV, 9 MeV
Linearity radiation intensity	3, 10, 50, 100, 300, and 400 MU
Reproducible irradiation count	10 times
Dose rate	100, 200, 400 MU/min
Depth	0.1, 0.3, 0.5, 0.8, 1, 1.3, 1.5, 1.9, 2, 2.3, 2.5, 2.8, 3, 3.3, 3.5, 3.8, 4, 6, 7, 9 cm
Source-to-surface distance	100 cm
Field size	14 cm × 14 cm (14 cone)

### Measurement setup

In this study, reproducibility and linearity were evaluated to confirm the precision and accuracy of the sensor. Additionally, the dose-rate dependence and PDI were evaluated to analyze the response characteristics of the radiotherapy QA procedure. For reproducibility measurements, all the sensors were irradiated 10 times. Then, to evaluate the response characteristics according to repeated irradiation, the measurements were normalized based on the signal obtained from the first beam.

In general, reproducibility represents the degree of scattering between the repeat measurements conducted under the same conditions and can be expressed using the relative standard deviation (RSD), which is derived as follows:

$$RSD(\%)=[\{\sum {\left(X_{i}-X_{Ave}\right)}^{2}/n{\}}^{0.5}/X_{Ave}]\times 100,$$ (1)

where X_i_ and X_Ave_ represent the response signal and mean response signal measured using the detector, respectively, and n represents the number of measurements. To compare the reproducibility of detectors comprising individually prepared mixtures, an RSD analysis was conducted. The evaluation criterion was that the detector precision must be within an RSD value of 1.5% at a 95% confidence level [15–17]. In the case of linearity evaluation, the dose was gradually increased to 3, 10, 50, 100, 200, 300, and 400 MU and evaluated with respect to the coefficient of determination (R^2^), as shown by the linear regression analysis. In this case, the evaluation criterion was set to R^2^ ≥ 0.9990. Accordingly, a sensor with high response stability and accuracy was selected based on the reproducibility and linearity evaluation results, with the dose-rate dependence and PDI evaluated subsequently. To evaluate the dose-rate dependence, doses of 50, 100, 200, and 400 MU were irradiated for the dose-rate settings of 100, 200, and 400 MU/min. The measured signal was normalized based on a dose rate of 200 MU, and the RSD (n = 3) for the value measured at 100 MU was calculated following the method reported in the diode study, and evaluated based on the reported diode error of 1.1% [18,19]. At this time, the results of the diode were as in [18], which was conducted under similar evaluation conditions to this study.

In the case of PDI evaluation, the dose was measured at depths of 0.1–9 cm through the slab phantom. Then, to calculate the percentage, the measurements were normalized according to the d-max point and compared with the thimble chamber (TM31010, PTW, Freiburg, Germany) result based on R_50_ [20].

The R_50,dos_ was determined by means of the measured PDI curves. The R_50,dos_ value is based on the distance between the water surface and the point beyond the dose maximum at which the PDI has a value of 50% (R_50,ion_) [21].

$$R_{50,dos}=1.029\;R_{50,ion}-0.06\;cm(R_{50,ion}\leq 10\;cm)$$ (2)

$$R_{50,dos}=1.059\;R_{50,ion}-0.37\;cm(R_{50,ion}>10\;cm)$$ (3)

Additionally, the R_50,dos_ can be determined from the percentage depth dose (PDD) curve after the PDI is converted to the PDD. This is achieved by multiplying the PDI with the mass stopping power ratio s_w,air_ according to the method described by Andreo et al. [20]. In this study, we compared the R_50,dos_ results with a thimble chamber using the PDI conversion method.

## Results

### Reproducibility

**Fig 2:**
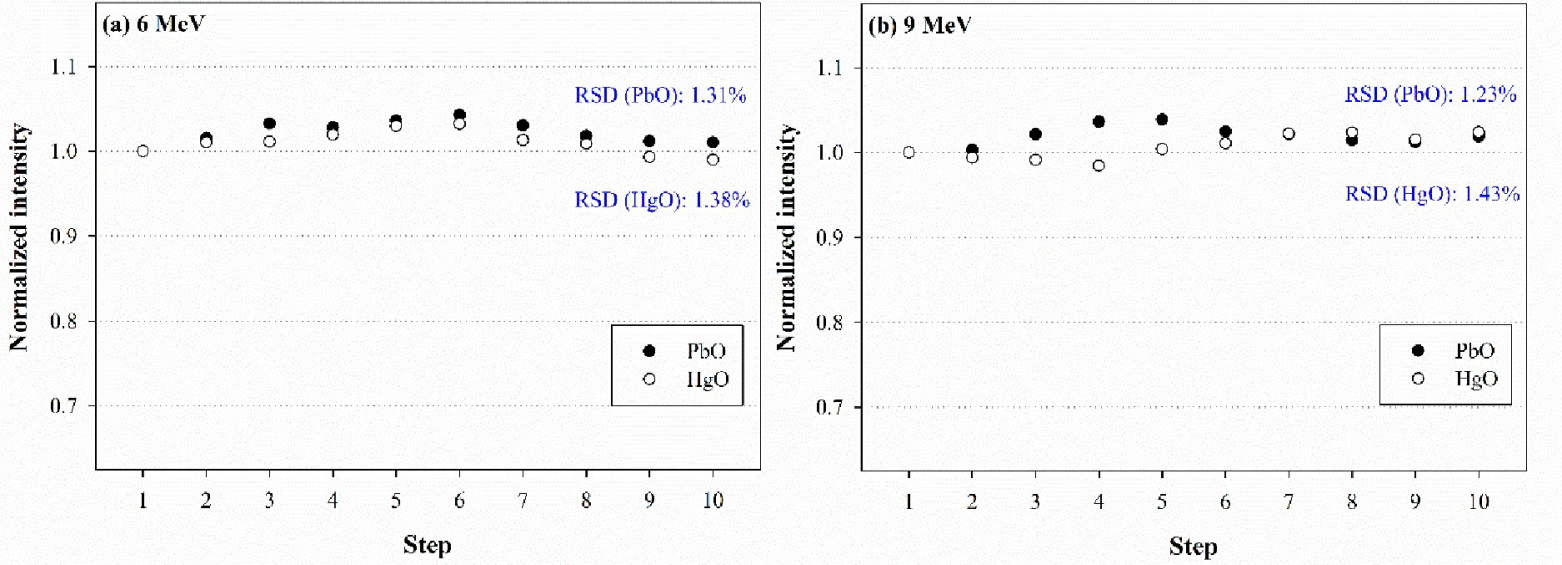
Reproducibility of each dosimeter at (a) 6 MeV and (b) 9 MeV.

The reproducibility and linearity were analyzed to evaluate the performance of flexible dosimeters based on PbO and HgO as monoxide photoconductors. Fig 2 shows the reproducibility results for each dosimeter.

For irradiation at 6 MeV, the analysis yielded RSD values for PbO and HgO of 1.31% and 1.38%, respectively, with corresponding values of 1.23% and 1.43% at 9 MeV. Accordingly, both sensors satisfied the requirement of RSD ≤ 1.5%. The PbO dosimeter outperformed the HgO dosimeter in terms of its stability characteristics by 0.07% at 6 MeV and 0.20% at 9 MeV.

### Linearity

**Fig 3:**
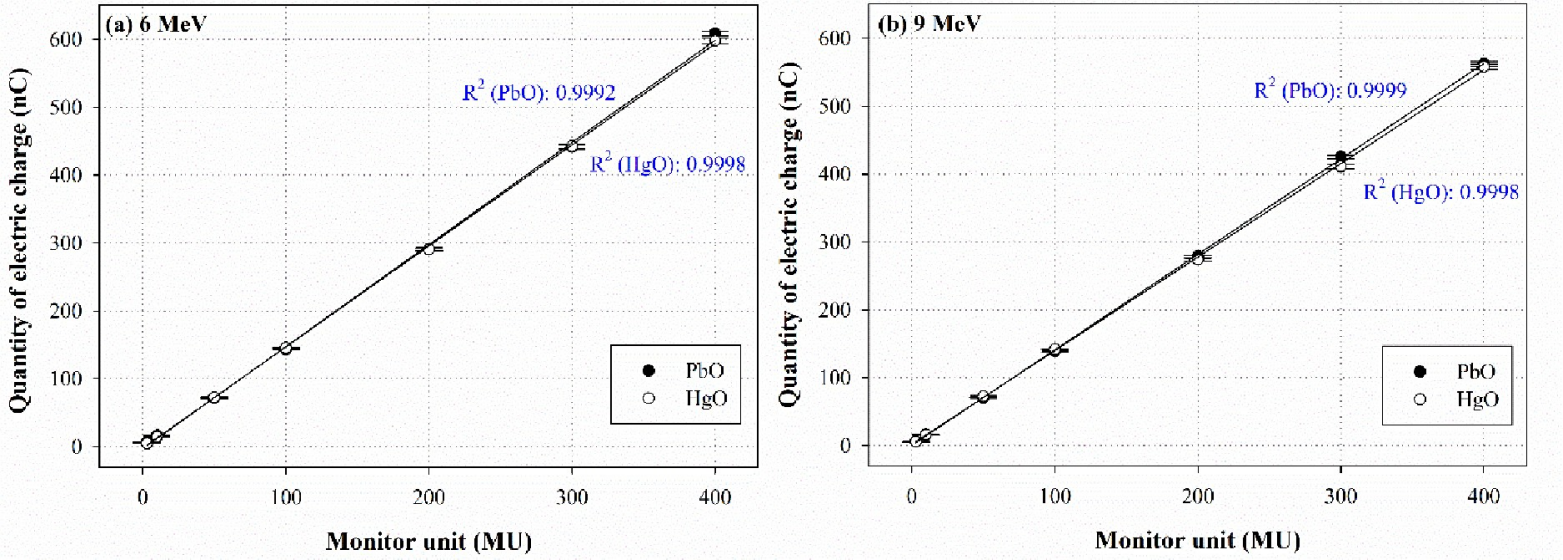
Linearity of each dosimeter at (a) 6 MeV and (b) 9 MeV.

We evaluated the linearity for each dosimeter to confirm the variation in EBT accuracy according to the irradiation dose, as shown in Fig 3.

For irradiation at both 6 and 9 MeV, both dosimeters demonstrated excellent linearity, with R^2^ ≥ 0.9990. Sensitivity is defined as the amount of charge (Q) generated per unit dose (D) corresponding to the linear function *a*, and has the following formula:

Q=aD+b (4)

Accordingly, the PbO and HgO sensitivities, as determined by the slope of the linear function *a*, were 1.504 and 1.489 at 6 MeV and 1.406 and 1.379 at 9 MeV, respectively. Based on these values, the PbO sensor was evaluated to be relatively superior to the HgO sensor.

### Dose-rate dependence

**Fig 4:**
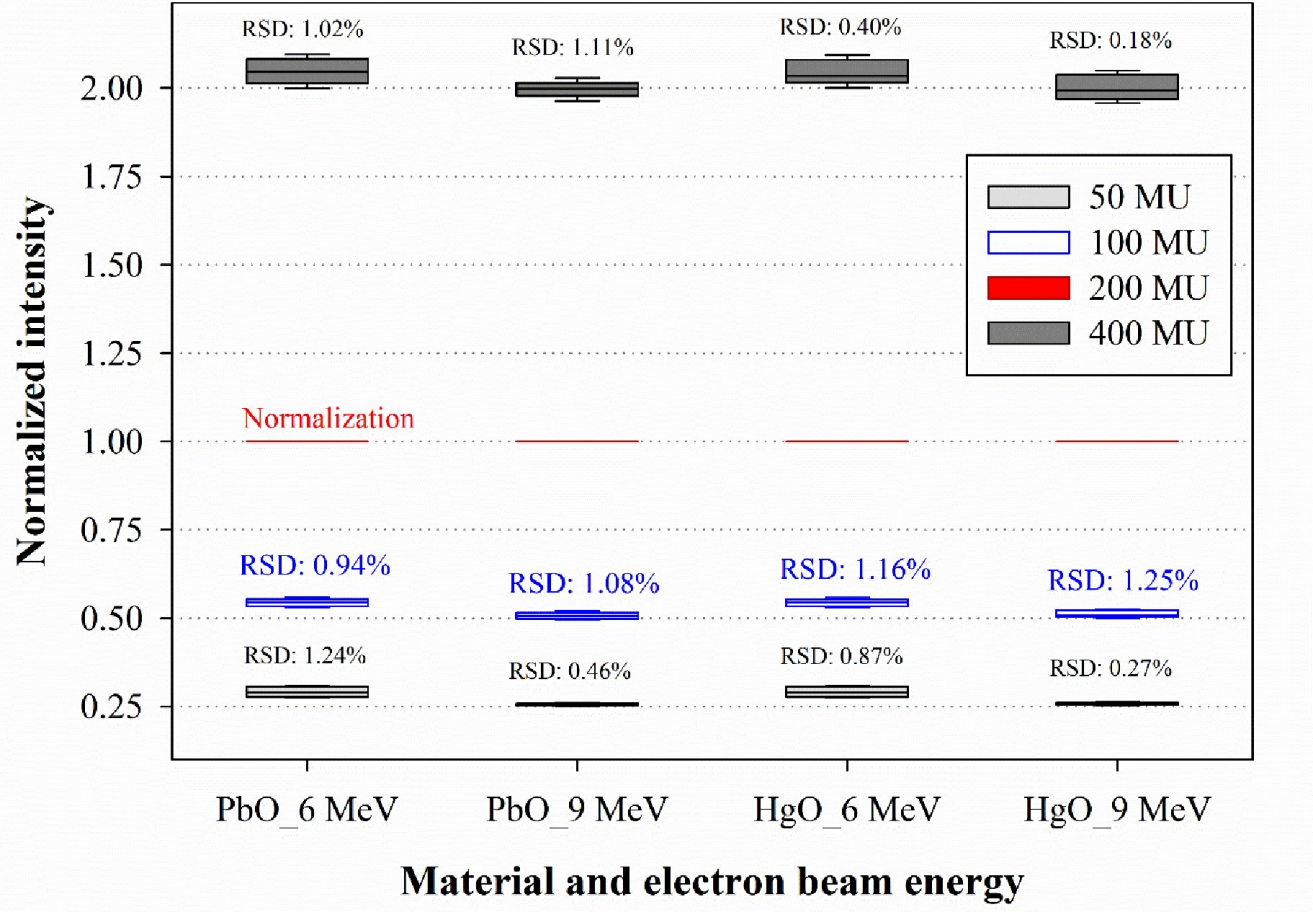
Dose-rate dependence of the PbO and HgO dosimeters at 6 MeV and 9 MeV electron beam energies.

Next, we evaluated the dose-rate dependence for the PbO and HgO dosimeters. Fig 4 shows the intensity error and RSD for each dose rate.

For the PbO dosimeter, the RSD for each dose-rate was calculated as 0.94% at 6 MeV and 1.08% at 9 MeV based on a dose of 100 MU. According to previous studies, the diode was reported to have an RSD of approximately 1.1% under the same conditions [18]. Compared with the diode, the PbO dosimeter presented here shows excellent characteristics, as evidenced by the lower RSD. For the HgO dosimeter, the RSD was determined as 1.16% at 6 MeV and 1.25% at 9 MeV, under the same conditions adopted for the PbO dosimeter measurements. These results exceeded the evaluation criterion of 1.1% (i.e., the RSD reported for the diode), thus reflecting a poor performance in comparison with the PbO dosimeter.

### Percentage depth ionization

**Fig 5:**
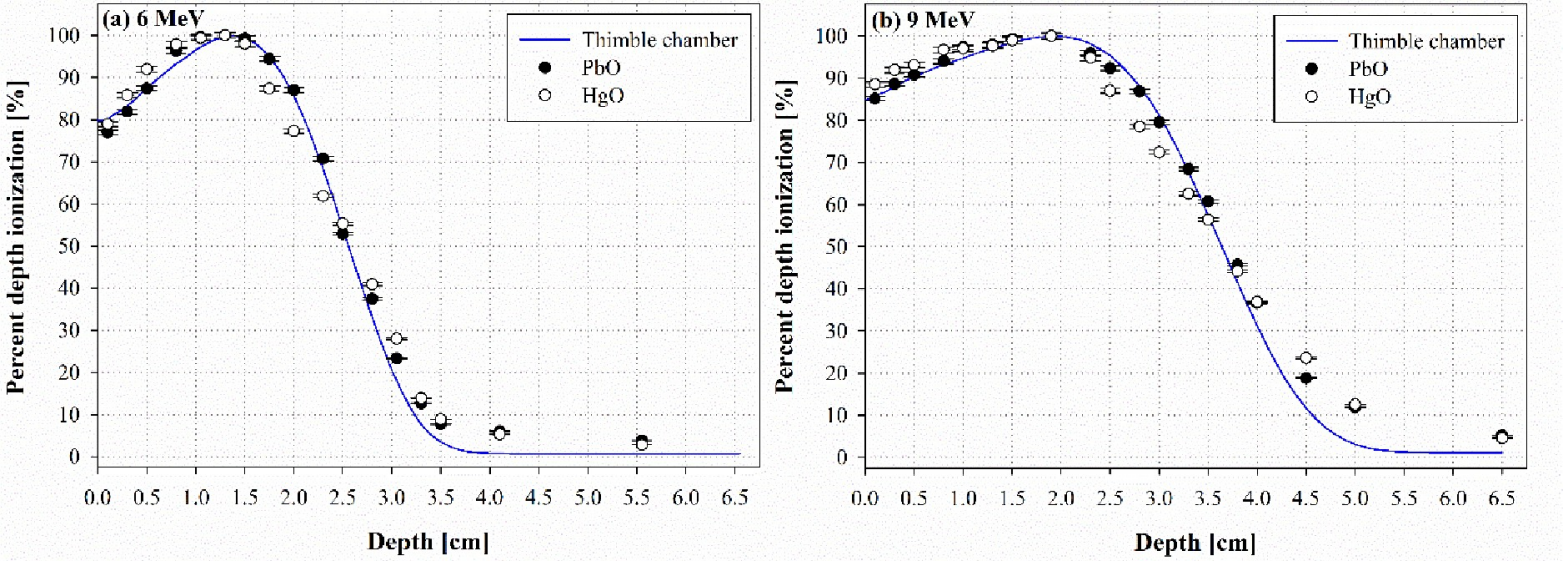
Percentage depth ionization of the dosimeters at (a) 6 MeV and (b) 9 MeV in comparison to that of a thimble chamber.

The PDI was obtained by conducting measurements at any depth between 0.1 and 6.5 cm. Fig 5 shows the PDI measurement for each dosimeter and a thimble chamber. Here, the value of the thimble chamber is measured by the dongnam institute of radiological and medical sciences of korea.

The PDIs of the PbO and HgO dosimeters were compared with their corresponding R_50,dos_ and R_80,dos_ values to evaluate their performance relative to that of the thimble chamber. At 6 MeV, the PbO and HgO dosimeters yielded R_50,dos_ values of 2.596 cm and 2.559 cm, respectively, which became 3.715 cm and 3.657 cm at 9 MeV, respectively. Compared with the R_50,dos_ values of the thimble chamber (6 MeV: 2.610 cm, 9 MeV: 3.641 cm), the R_50,dos_ values of the PbO and HgO dosimeters showed differences of 0.014 cm and 0.051 cm, respectively, at 6 MeV, and –0.074 cm and –0.016 cm, respectively, at 9 MeV.

At 6 MeV, the PbO and HgO dosimeters yielded R_80,dos_ values of 2.120 cm and 1.926 cm, respectively, which became 2.743 cm and 2.957 cm at 9 MeV, respectively. Compared with the R_80,dos_ values of the thimble chamber (6 MeV: 2.106 cm, 9 MeV: 2.952 cm), the R_80,dos_ values of the PbO and HgO dosimeters showed differences of –0.014 cm and 0.180 cm, respectively, at 6 MeV, and 0.209 cm and –0.005 cm, respectively, at 9 MeV. The overall mean errors of the PbO and HgO dosimeters were approximately –1.78% and –1.55% at 6 MeV, and –3.01% and –5.13% at 9 MeV, respectively, exhibiting a similar tendency to that of the chamber. Therefore, it was possible to measure 6 MeV electron beam quality with a semiconductor dosimeter having an error of less than ~2%.

## Discussion

This study analyzed the reproducibility and linearity of polycrystalline PbO and HgO dosimeters fabricated by the PIB deposition method, and further evaluated the dose-rate dependence and the PDI.

As a result of reproducibility evaluation, PbO and HgO dosimeters demonstrated an RSD within 1.5%, which satisfies the 95% confidence interval. Additionally, both dosimeters showed excellent linearity, indicated by their R^2^ values of 0.9998 or higher. The dose-rate dependence evaluation revealed that the HgO dosimeter underperformed compared with the diode standard (1.1%); however, the PbO dosimeter yielded a lower linearity according to dose rate of 1.04%. Therefore, the PbO dosimeter shows strong potential as a semiconductor dosimeter capable of replacing the diode.

The PDI results showed that when comparing the thimble chamber and R_50_ value, the maximum errors associated with the PbO and HgO dosimeters were 0.014 cm and 0.051 cm, respectively, at 6 MeV, and -0.074 cm and -0.016 cm, respectively, at 9 MeV. This difference may be caused by the air gap between the slab phantom and slab phantom, which is generated as the measurement depth increases. Based on the overall results, the PbO dosimeter exhibited superior performance properties compared to its HgO counterpart. According to Task Group 142, which is widely used as a recommendation for medical linear accelerator QA, it is recommended that the R_50_ value is within ± 1 mm for annual EBT QA [22]. Therefore, as the results of this study confirm the suitability of PIB-fabricated PbO dosimeters as electron dosimeters.

Owing to the simplicity of the PIB deposition method relative to the single-crystalline manufacturing method, the film-based polycrystalline monoxide dosimeters proposed here offer a distinct advantage in terms of production cost. Moreover, the accuracy of the dose verification will continue to improve with additional studies on the correction factors for each variable, such as energy dependence and dose-rate dependence.

However, the film semiconductor dosimeter proposed in this study does have its disadvantages. When attached to the human body, it can cause electric shock and substance toxicity problems—to prevent these problems, a protective layer must be considered. The physical properties, especially ductility, of the materials used for the composition of the protective layer, as well as of the dosimeter, should be considered, and the corresponding effect on the attenuation rate should be analyzed. Additionally, the applications of various passive layers, and the aging problem of metal oxides must be studied. These flexible digital dosimeters can be used as in vivo dosimeters, with multiple potential applications such as high energy cone beam CT, dental radiography, and radiographic testing. Electron beams contain various parameters, such as scattering in the beam path, irregular fields, and measurement thickness effect. Therefore, the determination of a pixel resolution that can analyze the two-dimensional (2D) dose distribution should be evaluated in future research.

## Conclusion

Image quality improvement is an important topic in the advancement of radiation detectors, with many studies exploring the potential of single- and polycrystalline materials for this purpose. However, in the field of radiation therapy, dosimeters have shown little progress with respect to measuring electron beams and no evaluation of treatment items has been conducted.

To address this issue, this study evaluated the performance of two polycrystalline monoxide semiconductor dosimeters based on a flexible functional material T-2 binder in terms of their suitability for measuring the skin dose of electron beams, which, to the best of our knowledge has not been evaluated previously. The study provides useful insights for the development of a flexible 2D dosimeter that can present skin dose distribution as a future dosimeter. Additionally, the QA evaluation aspect of this study can help guide future research directions for the development of an optimized dosimeter.

Flexible functional materials are promising materials capable of overcoming the morphological limitations of rigid materials and have a promising future in dosimeter development. Therefore, this study provides basic data for all radiation-based measurement fields.
